# The Us2 Gene Product of Herpes Simplex Virus 2 modulates NF-κB activation by targeting TAK1

**DOI:** 10.1038/s41598-017-08856-4

**Published:** 2017-08-21

**Authors:** Xuan Lu, Changjing Huang, Yi Zhang, Yong Lin, Xueyu Wang, Qian Li, Shi Liu, Jingfeng Tang, Li Zhou

**Affiliations:** 1 Department of Medical Genetics, Wuhan University School of Basic Medical Sciences, Wuhan, 430072 China; 2 Animal Biosafety Level III Laboratory at the Center for Animal Experiment, Wuhan University School of Medicine, Wuhan, 430072 China; 3 State Key Laboratory of Virology, College of Life Sciences, Wuhan University, Wuhan, 430071 China; 4 College of Biological Engineering and Food Sciences, Hubei University of Technology, Wuhan, 430068 China; 5 Institute of Virology, University Hospital of Essen, University of Duisburg-Essen, Essen, 45122 Germany

## Abstract

HSV-2 is one of the most common sexually transmitted pathogens worldwide and HSV-2 infection triggers cytokine and chemokine production. However, little is known about which HSV-2 genes engage in the regulation of NF-κB signaling and what mechanisms are involved. In a screen of the unique short (Us) regions of HSV-2, we observed that HSV-2 Us2 activates NF-κB signaling. We additionally indicated that deficiencies of Us2 decrease HSV-2 WT mediated NF-κB activation and cytokine and chemokine production, and overexpression of Us2 showed opposite effects. Co-immunoprecipitations indicated that Us2 interacted with TGF-β activated kinase 1 (TAK1), a serine/threonine kinase essential for NF-κB activation, and Us2 has the ability to regulate the TAK1-mediated pathway and induces TAK1 downstream signaling. Further studies verified that Us2 induced the phosphorylation of TAK1, resulting in the activation of TAK1 mediated downstream signaling. The role of Us2 in HSV-2 induced NF-κB pathways was also confirmed in the Us2-deficient mutant and HSV-2 WT infected mice. Our results indicate that HSV-2 Us2 gene product binds to TAK1 to positively regulate NF-κB signaling and, for the first time, provide insights into the molecular mechanism.

## Introduction

The transcription factor NF-κB plays a pivotal role in many cellular events such as apoptosis, cell proliferation, inflammation, and immunity^1, 2^. Generally, NF-κB is sequestered in the cytoplasm by the IκB proteins family of inhibitory proteins, resulting in the inactivation of NF-κB^3, 4^. A range of stimuli, including bacterial and viral infection, inflammatory cytokines, oxidative stresses, and the engagement of antigen receptors, leads to activation of the IkB kinase (IKK) complex. The activated IKK complex phosphorylates and ubiquitinates IkB proteins, releasing the bound NF-κB dimers to translocate to the nucleus where they can bind as dimers to κB sites in promoters to enhance a variety of genes^5–7^. The dysregulation of NF-κB activity is associated with metabolic diseases, cancer inflammatory disorders, and autoimmune activities^8^.

Given that the regulation of IKK activity is a key event in the activation of NF-κB, it is not surprising that IKK activity is tightly controlled at multiple levels by regulatory elements^9, 10^. Among these regulatory elements, the kinase TAK1 (transforming growth factor-β (TGF-β)–activated kinase 1) is the one of most important regulators^11^. TAK1 belongs to the MAPKKK family of regulators and mediates various intracellular actions of proinflammatory cytokines, such as TNF-α and interleukin 1β (IL-1β)^12^. TAK1 is essential in the communication of the upstream signal from the receptor complex to the downstream signaling molecules^13^. For example, the binding of IL-1β to its receptor IL-1R leads to interaction with the adaptor protein MyD88^14^. MyD88 recruits IRAK family members, which in turn activates TRAF6, a ubiquitin E3 ligase^14^. Ubiquitinated TRAF6 associates with TAB and TAK1 to form a complex, resulting in the activation of TAK1^15^. Once activated, TAK1 initiates a cascade of signaling events, including activation of the IKK complex, phosphorylation and ubiquitination of IkB, and the translocation of NF-κB to the nucleus^16^.

Herpes simplex virus type 2 (HSV-2) is a major sexually transmitted pathogen with a worldwide prevalence exceeding 500 million^17, 18^. HSV-2 can infect the genital epithelium to cause genital herpes and can also be transmitted to the central nervous system to establish a life-long latent infection^19, 20^. The innate response plays an important role in the HSV-2 life cycle. In the early stages of the infection, the virus is cleared from the epithelium by the immune response^21^. Later in infection, the virus manages to escape the immune responses to establish and maintain a latent infection^22^. As part of the host innate response, studies have shown that HSV-2 infection activates NF-κB signaling through Toll-like receptor (TLR) 9-dependent and TLR 9-independent pathways^23, 24^. Although HSV-2 induced NF-κB signaling has been described, the regulatory mechanism has not been fully established.

HSV-2 is a double-stranded DNA virus that is part of the Herpesviridae family, genus Simplexvirus. The DNA genomes of HSV-2 have at least 74 open reading frames and are divided into unique long (Ul) and unique short (Us) regions. We investigated if any of the HSV-2 Us regions play a role in activation of NF-κB. Our results show that HSV-2 Us2 is essential for HSV-2-induced NF-κB activation. Additionally, we demonstrated that Us2 interacts with TAK1 leading to activation of TAK1’s downstream genes. Our results describe a novel mechanism for NF-κB activation during HSV-2 infections.

## Results

### Us2 activates NF-κB signaling and induces the production of proinflammatory cytokines and chemokines

It is well established that HSV-2 infection activates NF-κB signaling through both TLR9 and non-TLR9 pathways^23, 24^. However, the regulatory mechanism of HSV-2-activated NF-κB signaling has not been adequately characterized. To screen the effect of the HSV-2 Us region for the ability to activate NF-κB reporter luciferase activity, we cloned the ORFs of the HSV-2 Us region and screened their ability to activate an NF-κB reporter gene in 293 T cells expressing TLR9. As shown in Supplementary Fig. 1A, the genes in the HSV-2 Us region, the Us2 gene product significantly stimulated NF-κB reporter luciferase activity. We also examined the effect of Us2 on NF-κB reporter luciferase activity in 293 T cells that did not contain the transfected TLR9 gene. Interestingly, Us2 also induced NF-κB reporter gene expression in a dose-dependent manner in this cell line (Supplementary Fig. 1B).

**Figure 1:**
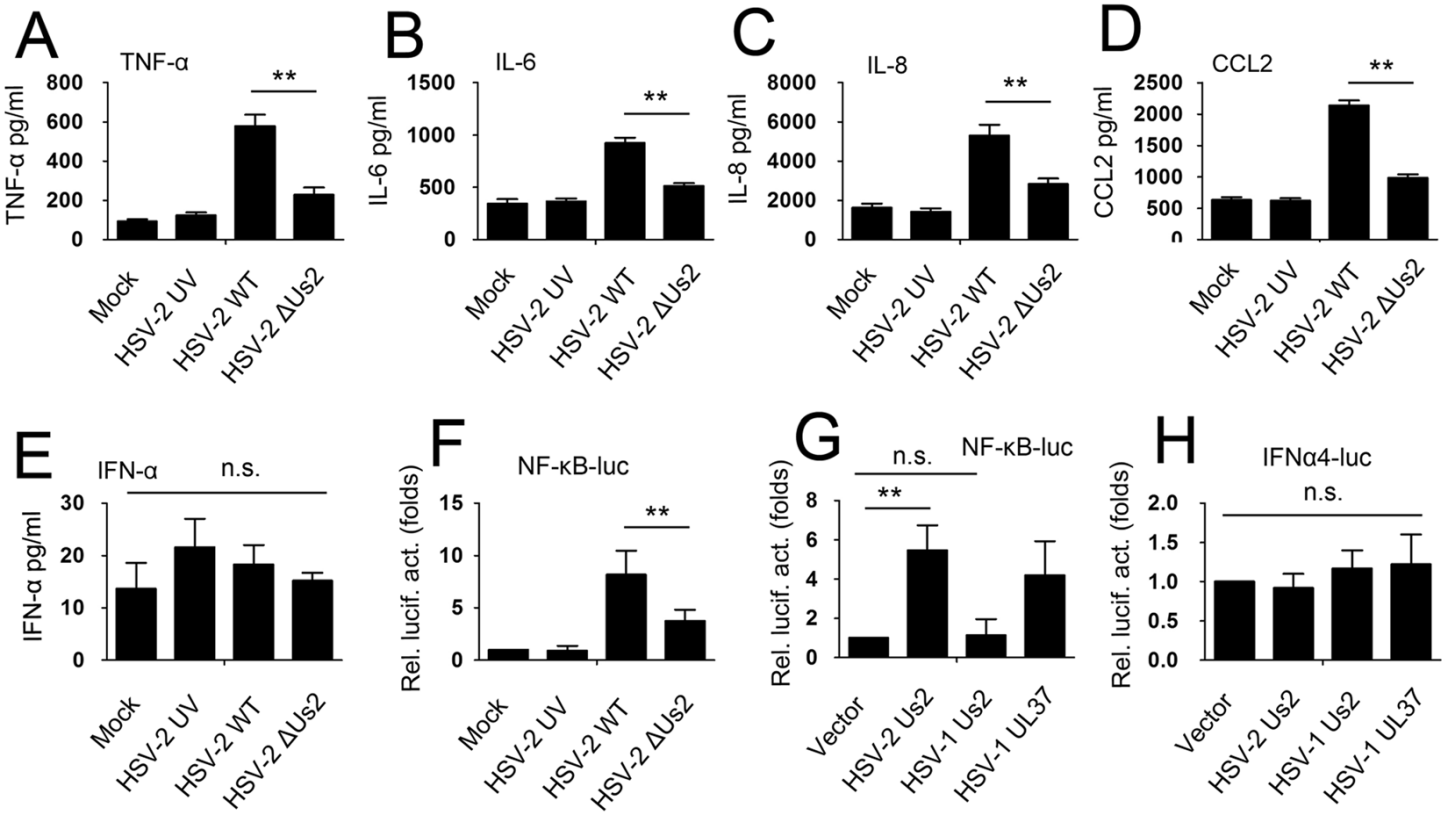
Effect of Us2 on NF-κB activity and proinflammatory cytokines and chemokines expression. (**A**–**E**) End1/E6E7 cells were infected with HSV-2 WT, HSV-2 UV, or HSV-2 ΔUs2 at 1 multiplicity of infection (MOI) for 12 h prior to ELISA analyses for TNF-α (**A**), IL-6 (**B**), IL-8 (**C**), CCL2 (**D**), IFN-α (**E**). (**F**) End1/E6E7 cells were transfected with NF-κB dualluciferase reporter plasmid (NF-κB-luc) for 12 h and infected with indicated viruses for 12 h prior to luciferase assays. (**G** and **H**) End1/E6E7 cells were transfected with the indicated plasmids for 24 h prior to luciferase assays. The results are the mean ± SD of triplicate cultures, representing three independent experiments (**P < 0.01).

To further investigate the role of Us2 on NF-κB activation, we constructed an Us2-deficient HSV-2 mutant as described previously^25^. HSV-2 was shown to induce two proinflammatory cytokines (TNF-α and IL-6) and two chemokines (IL-8 and CCL2) production, but not alter IFN-α production, in primary human genital epithelial cells^26^. These identified factors of TNF-α, IL-6, IL-8, CCL2, and IFN-α are all regulated by NF-κB. We next examined the effect of Us2 on expression of these proinflammatory cytokines and chemokines using an Us2-deficient HSV-2 mutant. As determined by ELISA analyses, HSV-2 WT infection strongly enhanced the production of TNF-α, IL-6, IL-8 and CCL2, but the levels of these proinflammatory cytokines and chemokines were inhibited in End1/E6E7 cells which were infected with the Us2 mutant of HSV-2 (Fig. 1A–D). There was no observed differences in IFN-α expression between the HSV-2 or the Us2 mutant infected cells (Fig. 1E). The effect of Us2 on proinflammatory cytokines and chemokines expression was not cell-type specific because similar results were observed in another human cervical epithelial cell line ME180 cells (Supplementary Fig. 2). We also explored the effect of HSV-2 and the Us2 mutant infection on NF-κB reporter gene expression. As shown in Fig. 1F, the deficiency of Us2 inhibited the HSV-2 induction of NF-κB reporter luciferase activity. We next investigated whether the HSV-1 Us2 gene similarly affected the activation of NF-κB reporter. Using the reporter assay, we found that the Us2 gene of HSV-1 did not stimulate NF-κB activation (Fig. 1G). It was previously reported that the UL37 gene of HSV-1 could stimulate NF-κB activation^27^, so transfection of UL37 was used as a positive control for activation. Additionally, neither expression of the Us2 gene of HSV-1 nor expression of the Us2 gene of HSV-2 induced the activity of the IFN-α promoter (Fig. 1H). These findings together indicate that HSV-2 Us2 plays a crucial role in NF-κB signaling.

### Us2 induced NF-κB signaling through TAK1

**Figure 2:**
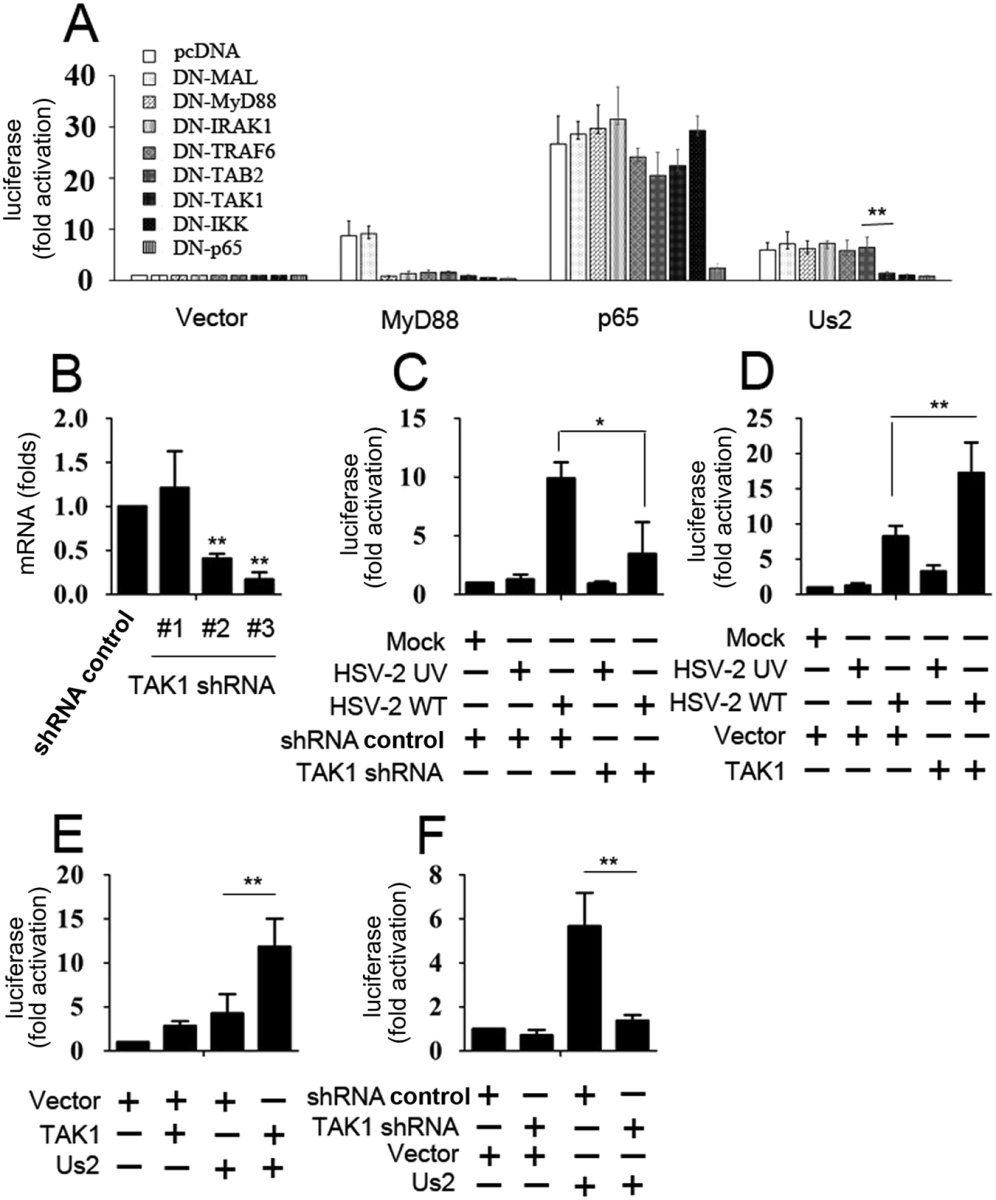
Identification of cellular signaling molecules needed for NF-κB activation by Us2. (**A**) 293 T cells were co-transfected with NF-κB-luc and dominant-negative mutant forms of MAL, MyD88, IRAK1, TRAF6, TAK1, TAB2, IKK, or p65 for 24 h prior to luciferase assays. (**B**) End1/E6E7 cells were transfected with the indicated TAK1 shRNAs for 48 h prior to real-time RT-PCR analyses. (**C**) End1/E6E7 cells NF-κB-luc and TAK1 shRNA or shRNA control for 12 h, and infected with the indicated viruses for 12 h prior to luciferase assays. (**D**) Experiments were performed as in C except that cells were transfected with pCMV-TAK1 or control vector. (**E** and **F**) 293 T cells were co-transfected with NF-κB-luc and the indicated plasmids for 12 h prior to luciferase assays. The results are the mean ± SD of triplicate cultures, representing three independent experiments (*P < 0.05, **P < 0.01).

To determine which signaling components in NF-κB signaling were required for Us2-induced signaling pathways, we constructed a panel of dominant-negative (DN) mutant forms of signaling intermediates including MAL, MyD88, IRAK1, TRAF6, TAB2, TAK1, IKK, and p65^27^. In reporter assays, overexpression of DN-p65 blocked the signaling induced by Us2, just as it blocked signaling by overexpression of MyD88 (Fig. 2A). Similarly, DN-TAK1 eliminated the activation of NF-κB reporter by Us2 but had no effect on p65 stimulation of NF-κB reporter activation (Fig. 2A). Overexpression of DN-MAL, DN-MyD88, DN-IRAK1, DN-TRAF6 or DN-TAB2 had no effect on Us2 signaling, but overexpression of DN-TAK1, DN-IKK, or DN-p65 inhibited Us2 activation of NF-κB (Fig. 2A). Thus, we speculated that Us2 regulates NF-κB signaling through TAK1. To confirm that TAK1 was needed for the Us2 activation of NF-κB, we designed three human TAK1 RNAi plasmids and tested their efficiency to alter Us2 activation (Fig. 2B). The plasmid expressing shRNA-TAK1 #3 was selected for the experiments described below. As shown in Fig. 2C and D, the knockdown of TAK1 dramatically decreased HSV-2-induced NF-κB activation and overexpression of TAK1 increased HSV-2-induced NF-κB activation. We next investigated whether Us2 is involved in the regulation of TAK1-mediated signaling. Results from luciferase activity assays indicated that TAK1 overexpression stimulated Us2-mediated activation of NF-κB and TAK1 expression knockdown inhibited Us2-mediated activation of NF-κB (Fig. 2E and F). Taken together, these results demonstrate that TAK1 plays an important role in Us2 induced NF-κB signaling.

### Us2 interacts with TAK1

**Figure 3:**
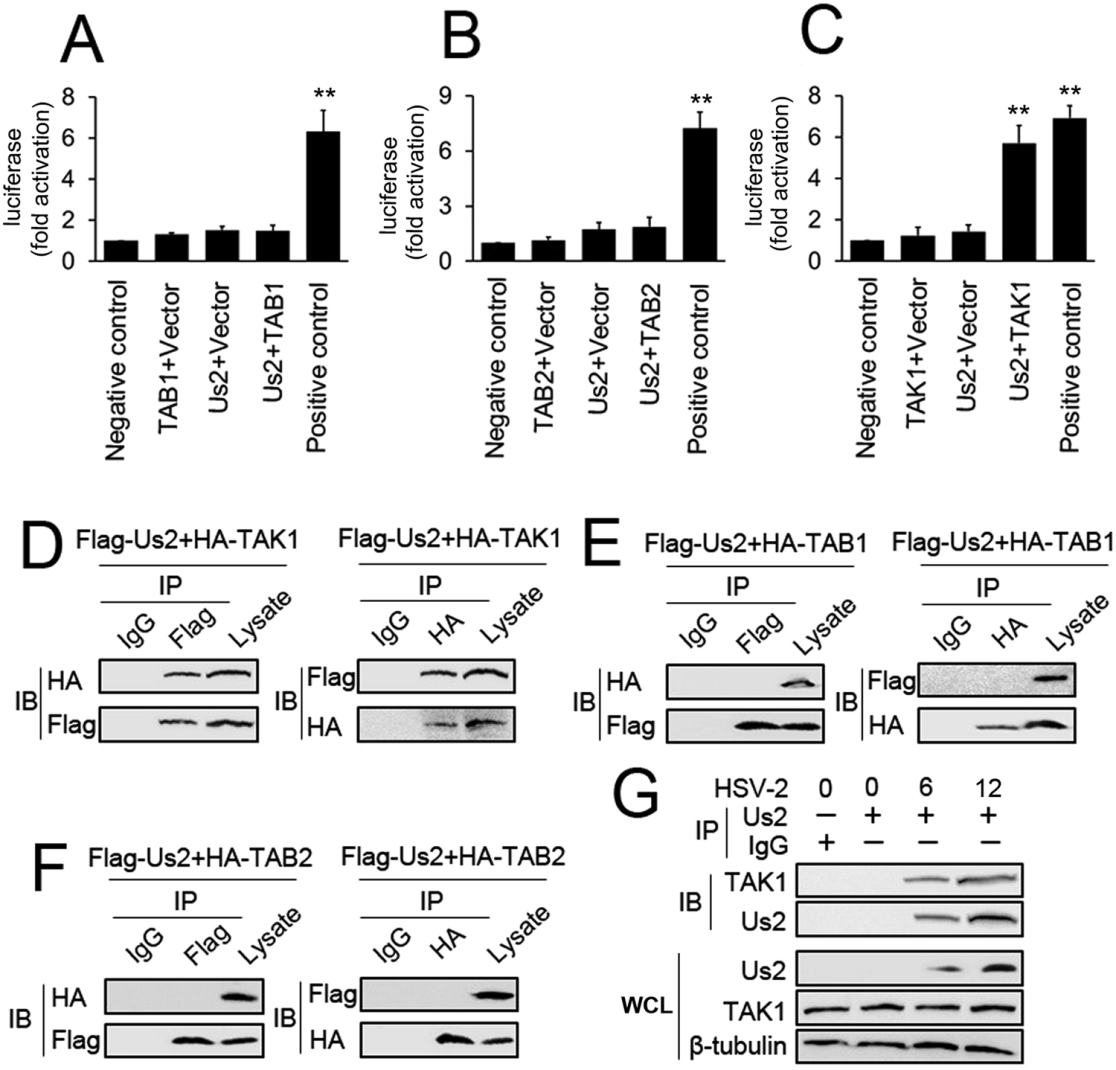
Detection of the interaction between Us2 and TAK1. **(A)** 293 T cells were co-transfected with pG5-luc (a luciferase reporter plasmid), pVP16-Us2, and pM-TAB1 or with control plasmids for 48 h prior to luciferase assays. (**B** and **C**) Experiments were performed as in A except that the 293 T cells were transfected with pM-TAB2 (**B**) or pM-TAK1 (**C**). (**D**) 293 T cells were transfected with Flag-tagged Us2 (Flag-Us2) and HA-tagged TAB1 (HA-TAB1). Forty-eight hours post-transfection, co-immunoprecipitation and immunoblot analysis were performed with the indicated antibodies. (**E** and **F**) Experiments were performed as in D except cells were transfected with HA-tagged TAB2 (HA-TAB2) (**E**) or HA-tagged TAK1 (HA-TAK1) (**F**). (**G**) End1/E6E7 cells were infected with HSV-2 for the indicated times or left uninfected. Immunoprecipitation and immunoblot analysis were performed with the indicated antibodies. All experiments were repeated at least three times with consistent results. Bar graphs represent the mean ± SD, n = 3 (*P < 0.05, **P < 0.01).

Given that the induction of NF-κB signaling by Us2 occurred mainly through TAK1, we hypothesized that Us2 interacts with the TAK1 complex. To test this hypothesis, we evaluated the binding of Us2 protein to TAB1, TAB2, or TAK1 *in vivo* using a GAL4/VP16-based mammalian two-hybrid system in 293 T cells. The results showed barely detectable luciferase activity in Us2 and the TAB1 or TAB2 co-transfection group (Fig. 3A and B). In contrast, the levels of luciferase activity in the Us2 and TAK1 co-transfection group were as high as in cells co-transfected with positive control plasmids (pM-p53 and pVP16-T; Fig. 3C). To confirm these results indicating interaction, we next performed transient transfection and co-immunoprecipitation experiments to further analyze the interaction between Us2 and TAK1. As shown in Fig. 3D, Flag-tagged Us2 interacted with HA-tagged TAK1. Reverse co-immunoprecipitation experiments also indicated that Flag-tagged Us2 interacted with HA-tagged TAK1 (Fig. 3D). In contrast, two other proteins in the TAK1 complex, TAB1 and TAB2, did not interact with Us2 (Fig. 3E and F). Interesting, HSV-1 UL37 and HSV-1 Us2 did not interact with TAK1 (Supplementary Fig. 3). We further performed endogenous co-immunoprecipitation experiments, and the results indicated that Us2 was weakly associated with TAK1 in cells infected with HSV-2 for 6 h; this association increased after 12 h of stimulation with HSV-2 (Fig. 3G). Because Us2 has previously been shown to associate with the plasma membrane (PM)^28^, we investigate the subcellular localization of Us2 with TAK1. In infected End1/E6E7 cells, Us2 and TAK1 localized to punctate cytoplasmic structures, to small vesicles that often appeared associated with the PM by 6 h postinfection (Supplementary Fig. 4A). At 12 h postinfection, Us2 and TAK1 was found predominantly at the PM, in cytoplasmic vesicles, and also localized diffusely throughout the cytoplasm (Supplementary Fig. 4A). A membrane flotation assay was performed to determine whether Us2 and TAK1 was associated with membranes. As shown in Supplementary Fig. 4B, both Us2 and TAK1 floated with cellular membranes, while EGFP, as a negative control, was not enriched in the membrane-containing fractions. These results suggest that during HSV-2 infection, Us2 associated with TAK1 to positively regulate NF-κB signaling.

### Us2 activates TAK1 downstream genes

**Figure 4:**
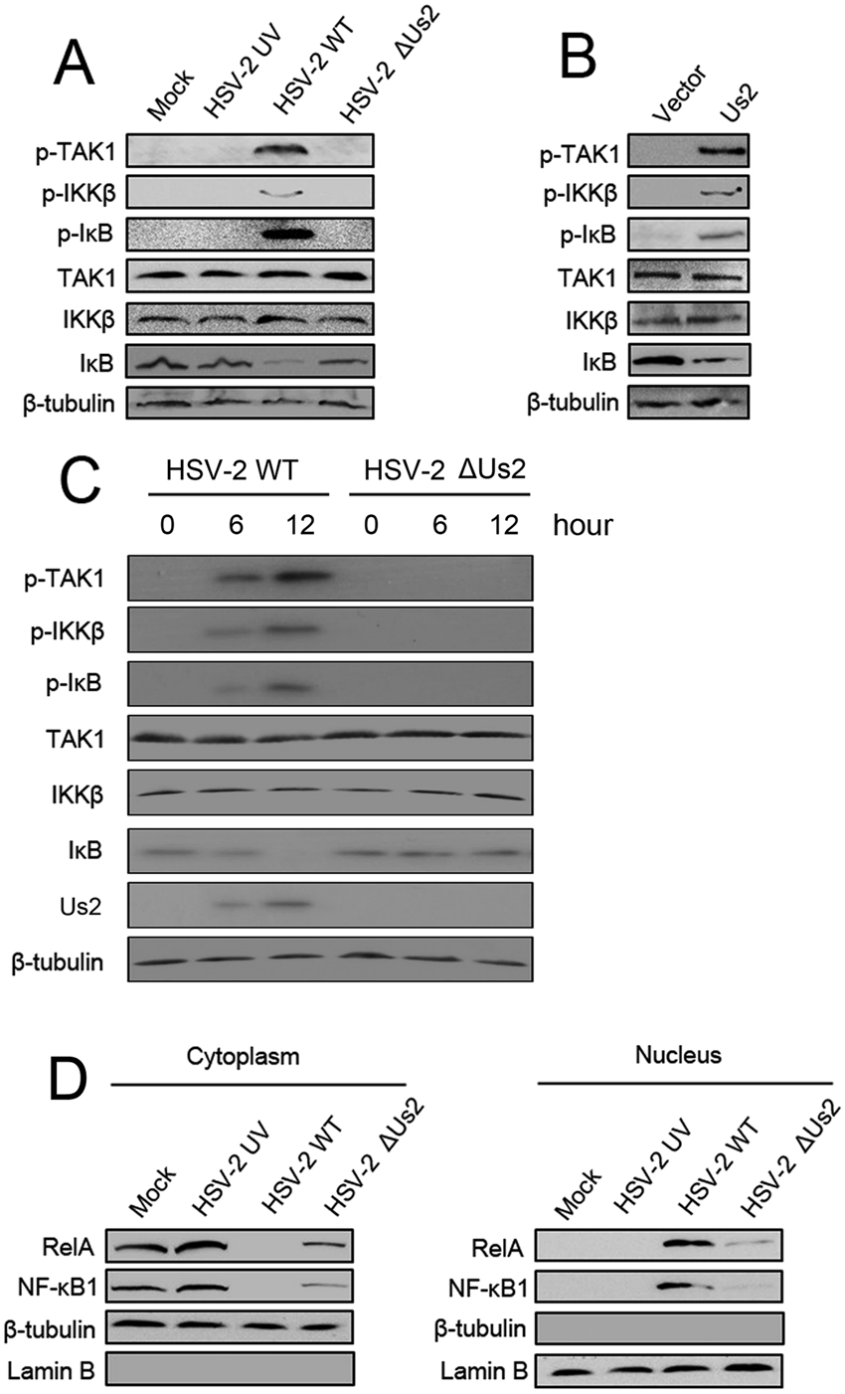
Us2 activates TAK1 and the downstream signaling pathways. (**A**) End1/E6E7 cells were infected with indicated viruses for 12 h prior to western blot analyses. (**B**) End1/E6E7 cells transfected with pCMV- Us2 or control vector for 24 h prior to western blot analyses. (**C**) End1/E6E7 cells were infected with HSV-2 WT or HSV-2 ΔUs2 for indicated times prior to western blot analyses. (**D**) End1/E6E7 cells were infected with the indicated viruses for 12 h. Cytosolic and nuclear extracts were prepared and subjected to western blot analyses. Lamin B and β-tubulin were used as markers for nuclear and cytosolic fractions, respectively. All experiments were repeated at least three times with similar results. Bar graphs represent the mean ± SD, n = 3 (*P < 0.05, **P < 0.01).

Activated TAK1 eventually phosphorylates and activates IKK leading to the phosphorylation and degradation of IκB proteins and thereby permitting the nuclear translocation of NF-κB^16^. To determine the role of Us2 on the TAK1-induced signaling pathway, End1/E6E7 cells were infected with HSV-2, UV-inactivated HSV-2, or the Us2 mutant of HSV-2. Western blot analyses showed that phoshporylated protein levels of TAK1, IKKβ, and IκB were increased in the presence of HSV-2 infection but decreased in the presence of the Us2 mutant infection; TAK1, IKKβ, and β-tubulin didn’t increase under all conditions (Fig. 4A). We also examined the effect of overexpression of Us2 on the TAK1/NF-κB pathway. Western blot experiments indicated that Us2 overexpression potentiated TAK1 and IKKβ phosphorylation and IκB phosphorylation and degradation (Fig. 4B). Further experiments showed that TAK1, IKKβ, and IκB phosphorylation was detected as early as 6 hours after HSV-2 WT infection (Fig. 4C). And phoshporylated protein levels of TAK1, IKKβ, and IκB were increased at 12 hours after HSV-2 WT infection (Fig. 4C). However, the Us2 mutant infection did not affect the phosphorylation status of TAK1, IKKβ, and IκB, suggesting that Us2 play an important role in NF-kB activation. We next examined the effect of Us2 on the translocation of NF-κB from the cytosol to the nucleus, a hallmark of production of proinflammatory cytokines and chemokines. As shown in Fig. 4D, NF-κB protein levels were lowered in the cytosol and elevated in the nucleus after HSV-2 infection. Infection with the Us2 mutant inhibited the translocation of NF-κB to the nucleus compared with HSV-2 infection. Similar results were obtained by immunofluorescence assays (Supplementary Fig. 5). Together, these results indicate that the TAK1-mediated signaling pathways were activated in response to Us2.

### Us2 potentiates production of proinflammatory cytokines and chemokines *in vivo*

**Figure 5:**
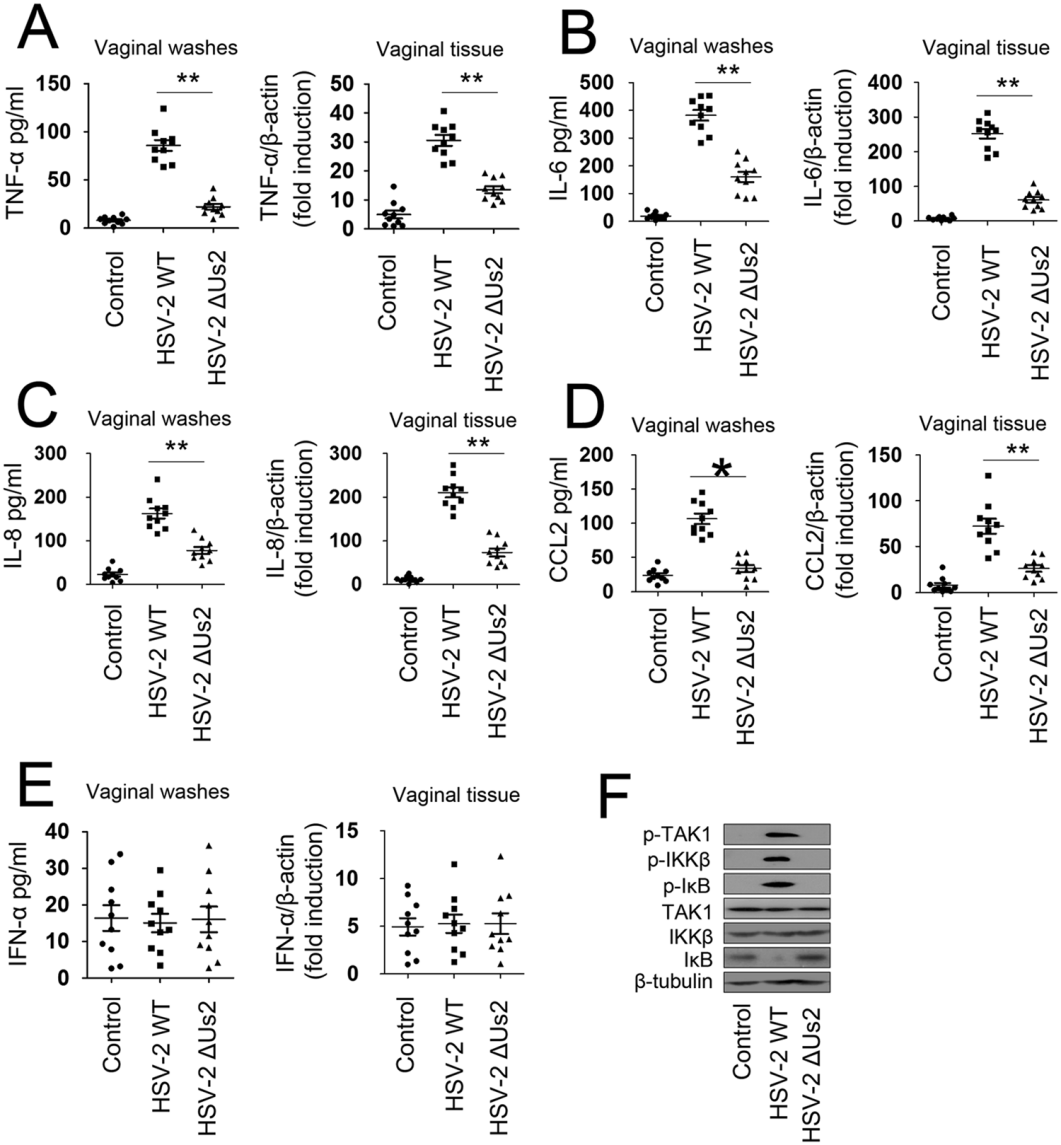
Us2 activates TAK1 and triggers an increase in production of proinflammatory cytokines and chemokines in mice. (**A**–**E**) Balb/c mice were infected intravaginally with medium (n = 10), 2 × 10^4^ PFU of HSV-2 WT (n = 10), or 2 × 10^4^ PFU HSV-2 ΔUs2 (n = 10) for 2 days prior to real-time RT-PCR (left panel) and ELISA (right panel) analyses for TNF-α (**A**), IL-6 (**B**), IL-8 (**C**), CCL2 (**D**) and IFN-α (**E**) in vaginal tissue (left panel) or vaginal washes (right panel). (**F**) Experiments were performed as in (**A**–**E)** except vaginal tissue was subjected to western blot analyses (n = 3). All data are graphed as mean values ± SEM (*P < 0.05, **P < 0.01).

Because Us2 plays an important role in HSV-2-induced production of proinflammatory cytokines and chemokines, we next explored whether Us2 regulated TAK1-mediated signaling pathways *in vivo*. As shown in Fig. 5A–D (left panel), there was a significant decrease in the concentrations of TNF-α, IL-6, IL-8, and CCL2 present in the vaginal washes following the Us2 mutant infection compared with HSV-2 infected mice. Similar results were obtained in excised tissue by using real-time RT-PCR assays (Fig. 5A–D, right panel). There were no statistically significant differences in the concentrations of IFN-α (Fig. 5E). Finally, we investigated whether Us2 plays a role in TAK1-mediated signaling pathways in vaginal tissue. In western blot assays, Us2 increased the phosphorylation of TAK1, IKKβ, and IκB (Fig. 5F). Overall, these results show that Us2 positively regulated TAK1-mediated signaling pathways in mice.

## Discussion

The HSV activation of NF-κB is biphasic with a first wave that is independent of viral replication and a second wave that requires viral gene expression^27^. However, the regulation mechanism for this activation of NF-κB by HSV-2 was unclear. In this study we determined that the Us2 gene product of HSV-2 plays an important role in NF-κB activation by interacting with TAK1.

Some infectious diseases can be a manifestation of a constant battle between the host and viruses^29^. Viral infection is sensed by the host innate immune system, which acts to limit viral infection by inducing antiviral cytokines^30^. To establish infection, many viruses have strategies to evade the innate immunity. Furthermore, in some cases viruses have evolved to use the host innate responses to their own advantages. In the innate immunity signal pathway using by viruses, NF-κB play an important role. For example, Human T-cell leukemia virus type 1 (HTLV-1) Tax enhances T cell growth and transformation via inducing NF-κB activation^31^. Epstein-Barr virus latent membrane protein 1 regulates B-lymphocyte growth transformation by inducing NF-κB signaling pathways^32^. Similarly, a growing body of evidence suggests that NF-κB activation increases the efficiency of HSV replication. HSV glycoprotein D abolished Fas-mediated apoptosis through NF-κB signaling from 1 to 2 h after induction^33^. HSV U_L_37 induce the NF-κB activation from 2 to 5 h after induction^27^. HSV ICP27 activated NF-κB signaling pathways after 5 h after induction^34^. In this study, we demonstrate that HSV-2 Us2 gene product positively regulate NF-κB signaling from 6 to 12 h after induction. In light of previous studies and our current results, gD and UL37 is response to early transient activation of NF-κB, while ICP27 and Us2 is response to the later activation of NF-κB. HSV-2 Us2 is a virion component that functions in the transport of the vesicles to the cell periphery, and its ability to induce NF-κB represents a possible second function.

We next investigate the role of HSV-2 Us2 on inflammatory cytokine production. Results shown that HSV-2 Us2 induced TNF-α, IL-6, IL-8 and CCL2 production (Figs 1 and 5). Interesting, a published work has been shown that HSV-2 Us2 is able to regulate IFN-γ production, but not IL-4 and IL-12 production^25^. Similar results were also observed in our study (Supplementary Fig. 6), implicating that Us2 can not regulate all inflammatory cytokine production. There are similar phenomena in other viruses. For example, Influenza A virus NS1 protein able to induce IL-1β and IL-18 production, but not IL-6 and MIP-1α^35^. Enterovirus 71 able to induce IL-1β, IL-6, IL-8, and IL-12p40 production, but not TNF-α production in human tonsillar epithelial cells UT-SCC-60A and UT-SCC-60B^36^. The reasons for this phenomenon need to be explored in future studies.

HSV-1 and HSV-2 share roughly 83% nucleotide identity in the protein coding regions and both can establish latency in sensory ganglia and recurrently reactivate to cause diseases^37^. However, HSV-1 and HSV-2 exhibit substantial differences in latency and reactivation patterns. In this study, our results show that HSV-2 Us2, which is conserved in most members of the *Alphaherpesvirinae* ^28^, induced the activation of NF-κB, but HSV-1 Us2 did not exhibit this enhancement effect (Fig. 1). Interestingly, a previous study has also found that, although HSV-1 Us1 shares nearly 60% of amino acid sequence with HSV-2 Us1, only HSV-2 Us1 inhibited IFN-β production^21^. Given that finding and our current results, we believe that despite the considerable homology (Supplementary Fig. 7), the HSV-1 and HSV-2 proteins exhibit distinct activities. To our knowledge, it may due to the difference in tertiary structure between HSV-1 and HSV-2 Us2. It may also due to the discrepancy between HSV-1 and HSV-2 infected site. Further studies will be needed to investigate these putative hypotheses.

To our knowledge, only one previous report has investigated the function of HSV-2 Us2. In that study, they reported that HSV-2 Us2 is a ubiquitin-interacting protein that is localized to the plasma membrane, recycling endosomes, and is also localized diffusely throughout the cytoplasm^28^. TAK1 is a serine/threonine protein kinase that functions as an important intermediate node in NF-κB signaling^13^. Some reports found that phosphorylation and polyubiquitination of TAK1 are involved in the regulation of the TAK1-mediated pathway^5^. Here, for the first time, we show that the Us2 protein interacts with TAK1 and Us2 overexpression induced the phosphorylation of TAK1 (Fig. 4). Because Us2 is an ubiquitin-interacting protein, Us2 also may have effects on the polyubiquitination of TAK1. Recently, several studies confirmed that HSV-2 induced TLR4-MyD88/Mal and IRAK1 signaling^22, 38^. TAK1 is a downstream signaling molecule of TLR4 and MyD88/Mal^39, 40^. Together with our work and previous studies, it seems like HSV-2 can regulate the function of proteins, which lies in one signaling pathway. Interestingly, there were other similar examples known. Enterovirus 71 3 C protein can inhibit RIG-I associated IFN response^41^. 3 C protein also can restrain this signaling pathway through cleavage of the TAK1/TAB1/TAB2/TAB3 complex^42^. HCV core protein blocks IFN signaling by interacting with signal transducers and activators of STAT1^43^. The core protein also induces expression of suppressor of cytokine signaling-1 (SOCS1) and SOCS3, and blocks Janus kinase–STAT signaling^44^. Our results suggest a mechanism that allows for virus to regulate inflammation network by interacting with proteins that play key roles inflammatory response.

**Figure 6:**
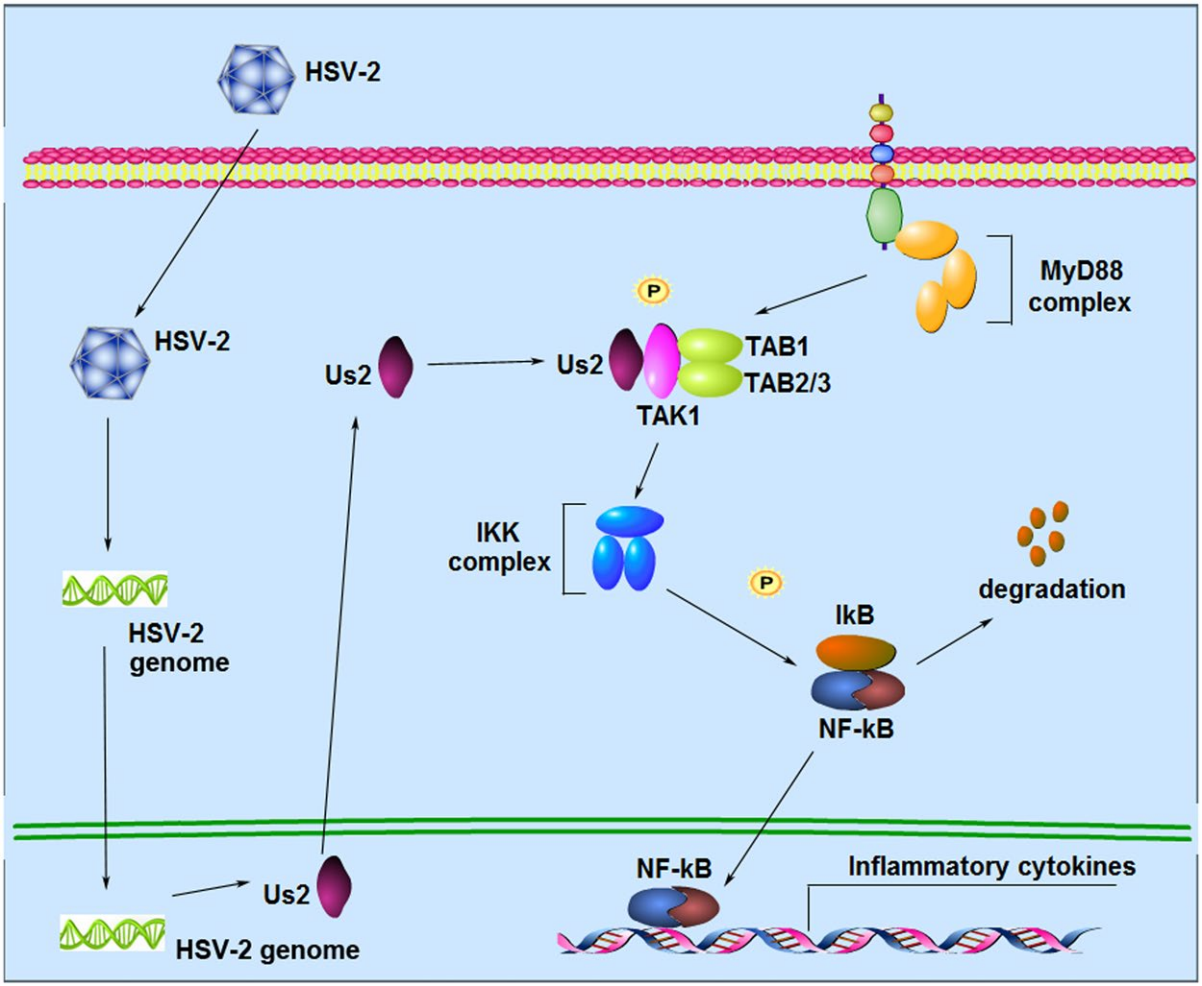
Schematic model of the role of Us2 in the regulation of NF-κB signaling. During HSV-2 infection, Us2 interacts with the kinase TAK1, which leads to activation of the IKK complex. The activated IKK complex induces phosphorylation and degradation of IκB, leading to translocation of NF-κB from the cytoplasm to the nucleus for the subsequent production of cytokines and chemokines.

Although other mechanisms may exist, we proposed a potential molecular model for Us2 regulation of NF-κB signaling (Fig. 6). During HSV-2 infection, HSV-2 Us2 interacts with TAK1 leading to the phosphorylation of TAK1, allowing it to activate the IKK complex. The IKK complex signals to translocate the NF-κB from the cytoplasm to the nucleus for subsequent production of proinflammatory cytokines and chemokines (Fig. 6). Although more studies are needed to understand the detailed regulatory mechanisms of Us2, our findings highlight the significance of HSV-2 Us2 in the production of proinflammatory cytokines and chemokines, providing a novel mechanism for Us2 in the regulation of NF-κB signaling.

## Materials and Methods

### Cell culture

Vero and 293 T cells were maintained in Dulbecco’s modified Eagle’s medium (DMEM) supplemented with 10% fetal bovine serum (FBS, Gibco, Grand Island, NY, USA). The human cervical epithelial cell line ME180 was cultured in McCoy’s 5 A modified medium (Gibco, Grand Island, NY, USA) supplemented with 10% FBS. The human End1/E6E7 cell line is a well-differentiated endocervical epithelial cell line immortalized by HPV 16 E6/E7 that is extensively studied as a human endocervical epithelial model^45–47^. The cells were cultured in keratinocyte growth medium (Gibco, Grand Island, NY, USA) supplemented with the provided recombinant epidermal growth factor (0.1 ng/ml) and bovine pituitary extract (50 μg/ml). All cells were grown at 37 °C in a 5% CO_2_ incubator.

### Viruses and reagents

The wild-type HSV-2 strain 186 and the Us2-deficient (YY2) was obtained from the State Key Laboratory of Virology (Wuhan University) and both viruses were generated as described previously^25^. The HSV-2 Us region expression plasmids were kind gifts of Dr. Yefu Wang, Wuhan University. NF-κB-luc plasmid were kind gifts of Dr. Qi Zhang, Wuhan University. Viruses were propagated on Vero cells and stored in aliquots at −80 °C until use. Viral titers were measured in Vero cells and expressed as plaque forming units (PFU)/ml and used for infection studies at a multiplicity of infection (MOI) of 1. UV-inactivated HSV-2 was obtained by exposure to UV irradiation for 20 min.

Antibody against β-tubulin and Lamin B were purchased from Santa Cruz Biotechnology (Santa Cruz, CA, USA). Human and mice antibodies against TAK1, p-TAK1, IKKβ, p-IKKβ, IκB, and p-IκB were purchased from Cell Signaling Technology (Beverly, MA, USA). Antibody against HSV-2 Us2 was purchased from WuXi AppTec (Shanghai, China). Antibody against Flag and HA were purchased from Sigma (St. Louis, MO, USA). All culture plasticware was obtained from Corning (Corning, NY, USA).

### Ethics Statement

8–10-week-old female BALB/c mice were purchased from the Center for Animal Experiment of Wuhan University School of Medicine. These animals were housed and handled at the Center for Animal Experiment of Wuhan University School of Medicine, which is certified by the Association for Assessment and Accreditation of Laboratory Animal Care International (AAALAC #001274). All housing and animal care procedures were in compliance with the China laws on animal experiments (Laboratory animal-Requirements of environment and housing facilities GB 14925-2010, China; Regulations on administration of laboratory animals, Ministry of Science and Technology, 1988, China) and with the 8^th^ Guide for the Care and Use of Laboratory Animals of Association (National Research Council, 2011). All the animal study protocols and procedure were approved by the Institutional Animal Care and Use Committee (IACUC) at the Center for Animal Experiment of Wuhan University School of Medicine prior to starting the experiment (AUP#2013127, experiment number S01314040T).

### Experimental animals

Mice were pretreated subcutaneously with 2.5 mg of medroxyprogesterone acetate (Sicor Pharmaceuticals, Irvine, CA) 5 days before intravaginal inoculation with HSV-2 delivered in 30 µl of PBS or PBS alone (control infection). Vaginal washes were collected in 150 µl of normal saline; mice were euthanized at specified times and the genital tract tissue was harvested for further analyses.

### Cytokine and chemokine measurements

Culture supernatants and vaginal washes were clarified by centrifugation (3000 rpm) for 5 min at 4 °C. The protein levels of tumor necrosis factor α (TNF-α), interleukin 6 (IL-6), interleukin 8 (IL-8), chemokine ligand 2 (CCL2), and interferon α (IFN-α) were measured using ELISA kits (eBioscience) according to the manufacturer’s instructions.

### RNA extraction and real-time RT-PCR

Total cellular RNA was extracted from cells using TRI-Reagent (Molecular Research Center, USA) according to the manufacturer’s instructions. Real-time RT-PCR was performed with IQ SYBR Green supermix (Bio-Rad Laboratories, Hercules, CA, USA) as described previously^48^. The levels of β-actin mRNA were used as an endogenous reference to normalize the quantities of target mRNA. Primers used in this study are listed in Supplementary Table 1 and were synthesized by Invitrogen Inc.

### Western blot

Whole-cell lysates were prepared using cell extraction buffer (Invitrogen, Shanghai, China) with 1% protease inhibitor cocktail (Sigma, MO, USA) according to the manufacturer’s instructions. Equal amounts of protein lysates (120 µg) were separated by 12% SDS-PAGE and transfected to polyvinylidene difluoride membranes (Millipore, Germany). Nonspecific sites were blocked with 5% nonfat dried milk before being incubated with Ab. Blots were developed with SuperSignal West Pico Chemiluminescent Substrate (Thermo Fisher Scientific, Waltham, MA, USA).

### Co-immunoprecipitation

Co-immunoprecipitation analysis was performed as previously described^49, 50^. Briefly, after cell treatment, cells were lysed using lysis buffer (50 mM Tris pH 8.0, 150 mM NaCl, 1% NP40, 1% protease inhibitor cocktail) at 4 °C. To eliminate the nonspecific binding of other proteins, the samples were pretreated with protein G-agarose beads for 3 h at 4 °C. Then, lysates were mixed and precipitated overnight at 4 °C with antibodies or IgG and protein G-agarose beads. Beads were washed five times with PBST and bound proteins were separated by 12% SDS-PAGE followed by western blot analysis.

### Nuclear extraction

To separate and collect the cytosolic and nuclear protein fractions, cells were washed twice with cold PBS and resuspended in 1 ml cold PBS, followed by centrifugation at 2000 rpm for 10 min in a microcentrifuge. The resulting pellets were resuspended in buffer A (1.5 mM MgCl_2_, 0.5% NP-40, 10 mM HEPES, pH 8, 10 mM KCl, 0.5 mM DTT, and 200 mM sucrose) for 10 min on ice with tube rotation. Nuclei were collected by centrifugation at 12,000 rpm for 15 s. Pellets were rinsed with buffer A, resuspended in buffer B (1.5 mM MgCl_2_, 420 mM NaCl, 20 mM HEPES, pH 7.9, 0.2 mM EDTA, and 1.0 mM DTT), and incubated on a rocking platform for 30 min (4 °C). Nuclei were clarified by centrifugation (12,000 rpm for 15 min). A cocktail of protease inhibitors was added to each type of buffer.

### RNA interference

TAK1 shRNA and a shRNA control (shRNA-control) were synthesized by RiBo Biotech (GuangZhou RiBo Biotech). The target sequence is shown in Supplementary Table 2.

### Mammalian two-hybrid analysis

293 T cells were transfected with luciferase reporter plasmid pG5-luc (Promega Corp., Madison, WI, USA), test plasmids, negative control plasmids and positive control plasmids respectively. Forty-eight hours post transfection, cells were harvested and luciferase activities were assayed by using the luciferase reporter assay system (Promega Corp., Madison, WI, USA) according to the manufacturer’s recommendations.

### Transfection and luciferase reporter gene assays

Cells were seeded on 24-well or 6-well dishes depending on the experiment, and were grown to 80% confluence prior to transfection. Cell were transfected using Lipofectamine 2000 (Invitrogen, USA) according to the manufacturer’s instructions. A Renilla luciferase reporter vector pRL-TK was used as an internal control. Luciferase assays were performed with a dual-specific luciferase assay kit (Promega Corp., Madison, WI, USA). Firefly luciferase activities were normalized on the basis of Renilla luciferase activities.

### Immunofluorescence

Cells were fixed with 4% paraformaldehyde for 15 min, washed three times with PBS and permeabilized with PBS containing 0.5% Triton X-100 for 5 min. Washed three times with PBS and blocked with PBS containing 4% BSA for 1 h at room temperature. Then, the cells were incubated with the primary antibody overnight at 4 °C, followed by incubation with Alexa492- labeled secondary antibodies (ProteinTech Group) for 1 h. Mounting was done with vectashield mounting medium with DAPI (Vector Laboratories), and the cells were visualized by confocal laser microscopy (FLUOVIEW FV1000; Olympus, Tokyo, Japan).

### Membrane flotation assays

The membrane flotation assays were performed as previously described^28^. In brief, cells were homogenized in 0.5 ml buffer (0.25 M sucrose, 10 mM KCl, 10 mM Tris-HCl (pH 7.4), 1.5 mM MgCl_2_) and incubated at 4 °C for 30 min. Next, cells were passed repeatedly through a 26-gauge syringe needle. Unbroken cells and nuclei and were removed by centrifugation (10 min). Then 0.3 ml of the postnuclear supernatant (PNS) was mixed with 2.7 ml of 85% (wt/vol) sucrose in NTE buffer (10 mM Tris-HCl (pH 7.4), 1 mM EDTA, 100 mM NaCl) and was placed in the bottom of an SW41 centrifuge tube. Next, 6 ml of 65% sucrose in NTE buffer, followed by 3 ml of 10% sucrose in NTE buffer, was layered onto the PNS. This sucrose step gradient was centrifuged at 100,000 × g for 20 h (4 °C). Fractions were collected from the top of the tube. Aliquots of fractions 1 to 12 were diluted with NTE buffer, and centrifuged at 100,000 rpm for 15 min to pellet the membranes. Pelleted material was resuspended in SDS-PAGE sample buffer. A cocktail of protease inhibitors was added to each type of buffer.

### Statistical analysis

Where appropriate, data were from at least three independent experiments and expressed as mean ± Standard deviation (SD). To compare the mean of two groups, the statistical significance was measured by Student’s t-test. To compare the difference between multiple groups, statistical significance was analyzed using a one-way analysis of variance followed by post Newman-Keul’s test. Calculations were performed with Graphpad Prism Statistical Software (GraphPad Software Inc, SanDiego, CA, USA). Statistical significance was defined as P < 0.05 or P < 0.01.

## Electronic supplementary material

Supplementary Information

## References

[1] Lawrence T (2009). The nuclear factor NF-kappaB pathway in inflammation. Cold Spring Harb Perspect Biol.

[2] Zheng C, Yin Q, Wu H (2011). Structural studies of NF-kappaB signaling. Cell Res.

[3] Liu S (2012). Major vault protein: a virus-induced host factor against viral replication through the induction of type-I interferon. Hepatology.

[4] Pomerantz JL, Baltimore D (2002). Two pathways to NF-kappaB. Mol Cell.

[5] Li Q (2011). Tripartite motif 8 (TRIM8) modulates TNFalpha- and IL-1beta-triggered NF-kappaB activation by targeting TAK1 for K63-linked polyubiquitination. Proc Natl Acad Sci USA.

[6] Newton, K. & Dixit, V. M. Signaling in innate immunity and inflammation. *Cold Spring Harb Perspect Biol* **4**, doi:10.1101/cshperspect.a006049 (2012). 10.1101/cshperspect.a006049 PMC3282411 22296764

[7] Vallabhapurapu S, Karin M (2009). Regulation and function of NF-kappaB transcription factors in the immune system. Annu Rev Immunol.

[8] Kawai T, Akira S (2011). Toll-like receptors and their crosstalk with other innate receptors in infection and immunity. Immunity.

[9] Shi M (2008). TRIM30 alpha negatively regulates TLR-mediated NF-kappa B activation by targeting TAB2 and TAB3 for degradation. Nature immunology.

[10] Wang C (2009). The E3 ubiquitin ligase Nrdp1 ‘preferentially’ promotes TLR-mediated production of type I interferon. Nature immunology.

[11] Fan YH (2011). USP4 targets TAK1 to downregulate TNFalpha-induced NF-kappaB activation. Cell Death Differ.

[12] Fan Y (2010). Lysine 63-linked polyubiquitination of TAK1 at lysine 158 is required for tumor necrosis factor alpha- and interleukin-1beta-induced IKK/NF-kappaB and JNK/AP-1 activation. The Journal of biological chemistry.

[13] Fan Y, Yu Y, Mao R, Zhang H, Yang J (2011). TAK1 Lys-158 but not Lys-209 is required for IL-1beta-induced Lys63-linked TAK1 polyubiquitination and IKK/NF-kappaB activation. Cell Signal.

[14] Beutler B (2004). Inferences, questions and possibilities in Toll-like receptor signalling. Nature.

[15] Gui S (2015). Mir-302c mediates influenza A virus-induced IFNbeta expression by targeting NF-kappaB inducing kinase. FEBS Lett.

[16] Wang C (2001). TAK1 is a ubiquitin-dependent kinase of MKK and IKK. Nature.

[17] Looker, K. J., Garnett, G. P. & Schmid, G. P. An estimate of the global prevalence and incidence of herpes simplex virus type 2 infection. *Bull World Health Organ* **86**, 805–812, A (2008). 10.2471/BLT.07.046128 PMC2649511 18949218

[18] Schiffer JT, Corey L (2013). Rapid host immune response and viral dynamics in herpes simplex virus-2 infection. Nat Med.

[19] Martinelli E (2011). HSV-2 infection of dendritic cells amplifies a highly susceptible HIV-1 cell target. PLoS Pathog.

[20] Wang K (2012). A herpes simplex virus 2 glycoprotein D mutant generated by bacterial artificial chromosome mutagenesis is severely impaired for infecting neuronal cells and infects only Vero cells expressing exogenous HVEM. Journal of virology.

[21] Zhang M (2015). HSV-2 immediate-early protein US1 inhibits IFN-beta production by suppressing association of IRF-3 with IFN-beta promoter. J Immunol.

[22] Liu H, Chen K, Feng W, Guo J, Li H (2014). HSV-2 increases TLR4-dependent phosphorylated IRFs and IFN-beta induction in cervical epithelial cells. PloS one.

[23] Lund J, Sato A, Akira S, Medzhitov R, Iwasaki A (2003). Toll-like receptor 9-mediated recognition of Herpes simplex virus-2 by plasmacytoid dendritic cells. J Exp Med.

[24] Sato A, Linehan MM, Iwasaki A (2006). Dual recognition of herpes simplex viruses by TLR2 and TLR9 in dendritic cells. Proc Natl Acad Sci USA.

[25] Inagaki-Ohara K, Iwasaki T, Watanabe D, Kurata T, Nishiyama Y (2001). Effect of the deletion of US2 and US3 from herpes simplex virus type 2 on immune responses in the murine vagina following intravaginal infection. Vaccine.

[26] Ferreira VH, Nazli A, Mossman KL, Kaushic C (2013). Proinflammatory cytokines and chemokines - but not interferon-beta - produced in response to HSV-2 in primary human genital epithelial cells are associated with viral replication and the presence of the virion host shutoff protein. Am J Reprod Immunol.

[27] Liu X, Fitzgerald K, Kurt-Jones E, Finberg R, Knipe DM (2008). Herpesvirus tegument protein activates NF-kappaB signaling through the TRAF6 adaptor protein. Proc Natl Acad Sci USA.

[28] Kang MH (2013). The Us2 gene product of herpes simplex virus 2 is a membrane-associated ubiquitin-interacting protein. Journal of virology.

[29] Levy DE, Garcia-Sastre A (2001). The virus battles: IFN induction of the antiviral state and mechanisms of viral evasion. Cytokine & growth factor reviews.

[30] Lutgehetmann, M. *et al*. Hepatitis B virus limits response of human hepatocytes to interferon-alpha in chimeric mice. *Gastroenterology* **140**, 2074–2083, 2083, e2071–2072, doi:10.1053/j.gastro.2011.02.057 (2011). 10.1053/j.gastro.2011.02.057 21376046

[31] Geleziunas R (1998). Human T-cell leukemia virus type 1 Tax induction of NF-kappaB involves activation of the IkappaB kinase alpha (IKKalpha) and IKKbeta cellular kinases. Molecular and cellular biology.

[32] Mosialos G (1995). The Epstein-Barr virus transforming protein LMP1 engages signaling proteins for the tumor necrosis factor receptor family. Cell.

[33] Medici MA (2003). Protection by herpes simplex virus glycoprotein D against Fas-mediated apoptosis: role of nuclear factor kappaB. The Journal of biological chemistry.

[34] Hargett D, Rice S, Bachenheimer SL (2006). Herpes simplex virus type 1 ICP27-dependent activation of NF-kappaB. Journal of virology.

[35] Stasakova J (2005). Influenza A mutant viruses with altered NS1 protein function provoke caspase-1 activation in primary human macrophages, resulting in fast apoptosis and release of high levels of interleukins 1beta and 18. The Journal of general virology.

[36] Xie GC (2016). Susceptibility of human tonsillar epithelial cells to enterovirus 71 with normal cytokine response. Virology.

[37] Kato A (2016). Roles of Us8A and Its Phosphorylation Mediated by Us3 in Herpes Simplex Virus 1 Pathogenesis. Journal of virology.

[38] Li H (2009). HSV-2 induces TLRs and NF-kappaB-dependent cytokines in cervical epithelial cells. Biochemical and biophysical research communications.

[39] Akira S, Takeda K (2004). Toll-like receptor signalling. Nature reviews. Immunology.

[40] Sato S (2005). Essential function for the kinase TAK1 in innate and adaptive immune responses. Nature immunology.

[41] Lei X (2010). The 3C protein of enterovirus 71 inhibits retinoid acid-inducible gene I-mediated interferon regulatory factor 3 activation and type I interferon responses. Journal of virology.

[42] Lei X (2014). Enterovirus 71 3C inhibits cytokine expression through cleavage of the TAK1/TAB1/TAB2/TAB3 complex. Journal of virology.

[43] Lin W (2006). Hepatitis C virus core protein blocks interferon signaling by interaction with the STAT1 SH2 domain. Journal of virology.

[44] Suda G (2010). IL-6-mediated intersubgenotypic variation of interferon sensitivity in hepatitis C virus genotype 2a/2b chimeric clones. Virology.

[45] Govender Y (2014). The injectable-only contraceptive medroxyprogesterone acetate, unlike norethisterone acetate and progesterone, regulates inflammatory genes in endocervical cells via the glucocorticoid receptor. PloS one.

[46] Hijazi K (2015). Expression of Genes for Drug Transporters in the Human Female Genital Tract and Modulatory Effect of Antiretroviral Drugs. PloS one.

[47] Sathe A, Reddy KV (2014). TLR9 and RIG-I signaling in human endocervical epithelial cells modulates inflammatory responses of macrophages and dendritic cells *in vitro*. PloS one.

[48] Zhou L (2015). Induction of interferon-lambda contributes to TLR3 and RIG-I activation-mediated inhibition of herpes simplex virus type 2 replication in human cervical epithelial cells. Mol Hum Reprod.

[49] Liu S (2015). Human hepatitis B virus surface and e antigens inhibit major vault protein signaling in interferon induction pathways. J Hepatol.

[50] Zhu SL (2016). Inducible CYP4F12 enhances Hepatitis C virus infection via association with viral nonstructural protein 5B. Biochemical and biophysical research communications.

