# Supplementary material for: The Us2 Gene Product of Herpes Simplex Virus 2 modulates NF-κB activation by targeting TAK1
Source: Sci Rep. 2017 Aug 21;7:8396. doi: 10.1038/s41598-017-08856-4 (PMC5566419; doi:10.1038/s41598-017-08856-4)
Supplement: Supplementary file 1 — Supplementary Information [file 41598_2017_8856_MOESM1_ESM.doc]

# SUPPLEMENTARY MATERIAL

**The Us2 Gene Product of Herpes Simplex Virus 2 modulates**

**NF-κB activation through targeting TAK1**

Xuan Lu ^1, 2, 3^, Changjing Huang ^4^, Yi Zhang ^4^, Yong Lin ^5^, Xueyu Wang ^5^, Qian Li ^5^, Shi Liu ^3^, Jingfeng Tang ^4^, Li Zhou ^2, *^

^1^ Department of Medical Genetics, Wuhan University School of Basic Medical Sciences, Wuhan 430072, China

^2^ Animal Biosafety Level III Laboratory at the Center for Animal Experiment, Wuhan University School of Medicine, Wuhan 430072, China

^3^ State Key Laboratory of Virology, College of Life Sciences, Wuhan University, Wuhan 430071, China

^4^ College of Biological Engineering and Food Sciences, Hubei University of Technology, Wuhan 430068, China

^5^ Institute of Virology, University Hospital of Essen, University of Duisburg-Essen, Essen 45122, Germany

**Supplemental Figure legends**

**Supplemental Figure 1.** Screening of HSV-2 Us region for activation of NF-κB. (**A**) 293T cells were transfected indicated plasmids for 24 h prior to luciferase assays. (**B**) 293T cells were transfected with NF-κB reporter (0.01 μg) and an increased amount of Us2 expression plasmid. Luciferase assays were performed 24 h after transfection. The results are mean±SD of triplicate cultures, representing three independent experiments (**P < 0.01; *P < 0.05).

**Supplemental Figure 2.** Us2 is essential for HSV-2 mediated NF-κB activation in human cervical epithelial cell line ME180 cells. (**A-E**) Experiments were performed as in Fig 1 A-E except ME180 cells were used. The results are mean±SD of triplicate cultures, representing three independent experiments (*P<0.05, **P<0.01), n.s., not significant.

**Supplemental Figure 3.** HSV-1 UL37 and HSV-1 Us2 did not interact with TAK1. (**A and B**) Experiments were performed as in Fig 3D except that the 293T cells were transfected with Flag-UL37 (HSV-1) (A) or Flag- Us2 (HSV-1) (B). All experiments were repeated at least three times with consistent results.

**Supplemental Figure 4.** Subcellular localization of HSV-2 Us2 and TAK1. **(A)** End1/E6E7 cells were infected with HSV-2 for indicated time prior to immunofluorescence assays. (B) 293T cells were cotransfected with pCMV- Us2, pCMV-TAK1 and EGFP expression plasmid. At 24 h after transfection, membrane fractions were isolated by membrane flotation on discontinuous sucrose step gradients. Fractions from the top (10% sucrose) to the bottom (71.5% sucrose) of the step gradient were analyzed by Western blotting. All experiments were repeated at least three times with similar results.

**Supplemental Figure 5.** Us2 induced the translocation of NF-κB from the cytosol to the nucleus. (**A and B**) Experiments were performed as in Fig 4C, except immunofluorescence assays were performed. Right: Localization of Rel A (A) and NF-κB (B) fluorescence was determined in 300 cells for each culture. The ordinate shows the percentage of cells with localization of Rel A (A) and NF-κB (B) mainly in the cytoplasm (white columns) or nucleus (black columns). The results are mean±SD of triplicate cultures, representing three independent experiments (*P<0.05, **P<0.01)

**Supplemental Figure 6.** Us2 did not affect IL-4 and IL-12 production. (A) Experiments were performed as in Fig 1A, except IL-4 and IL-12 production were analyzed. (B) Experiments were performed as in Fig 5A, except IL-4 and IL-12 production were analyzed. All data are graphed as mean values±SEM (*P<0.05, **P<0.01, n=5). n.s. not significant.

**Supplemental Figure 7.** Analyze the protein sequence of HSV-1 and HSV-2 Us2.

**Supplemental Figure 1**

**
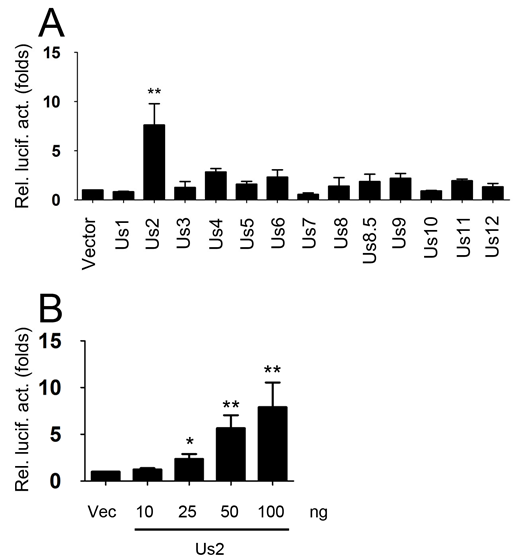
**

**Supplemental Figure 2**


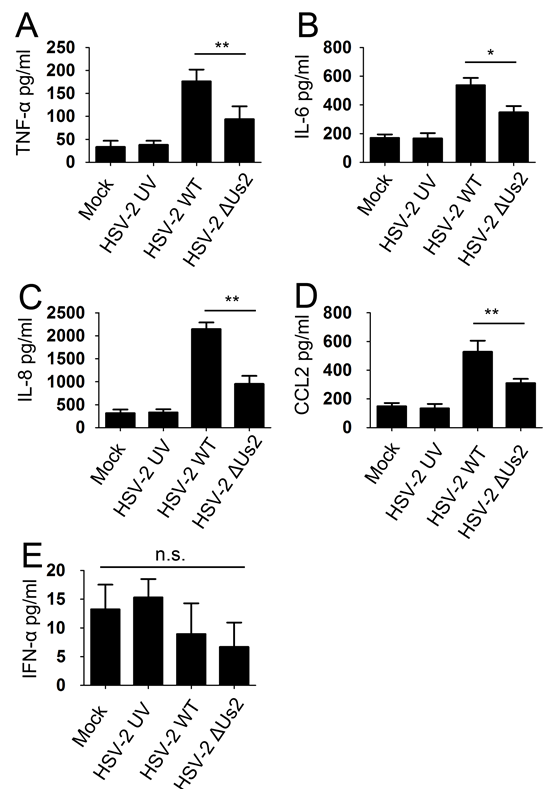


**Supplemental Figure 3**


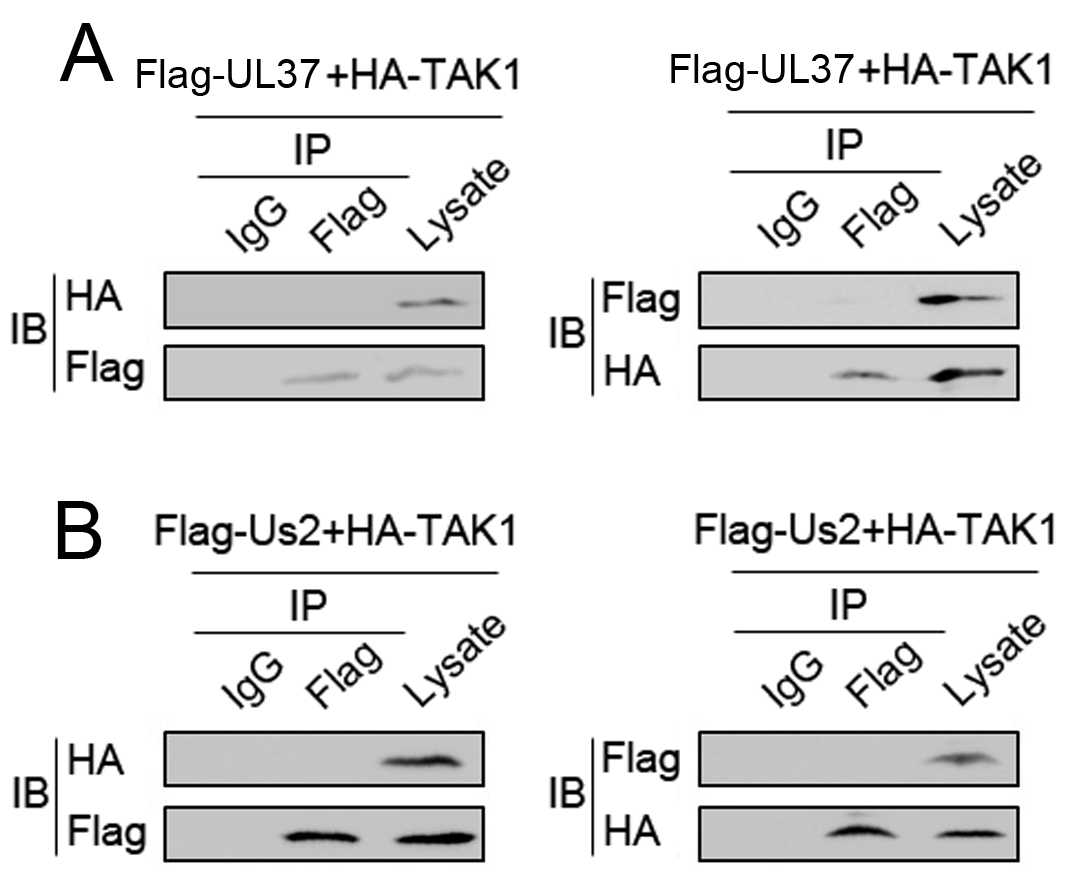


**Supplemental Figure 4**


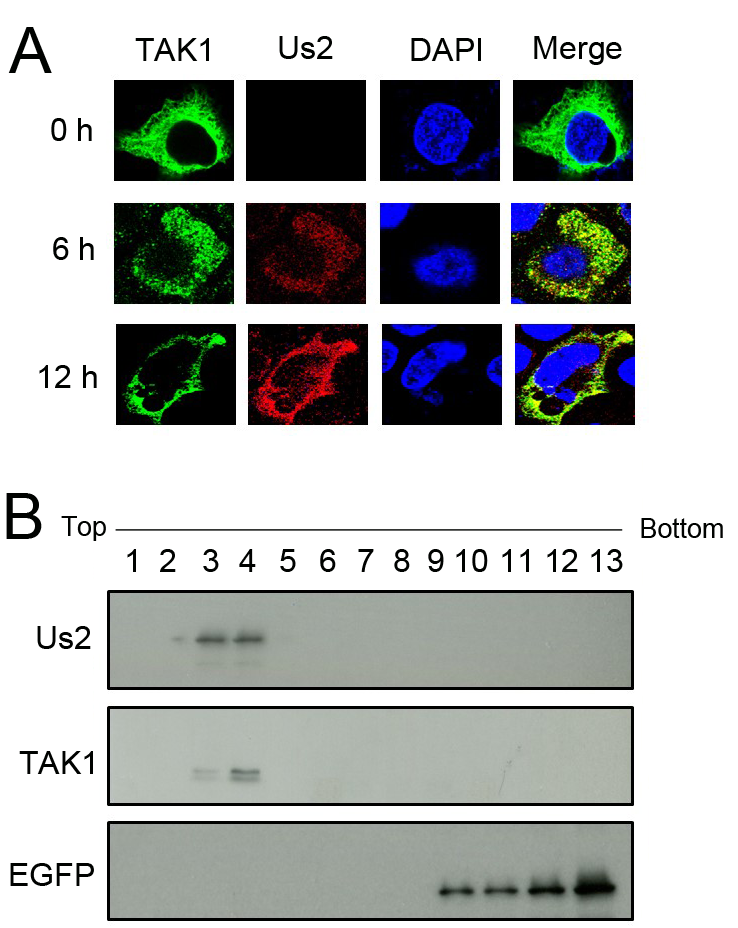


**Supplemental Figure 5**


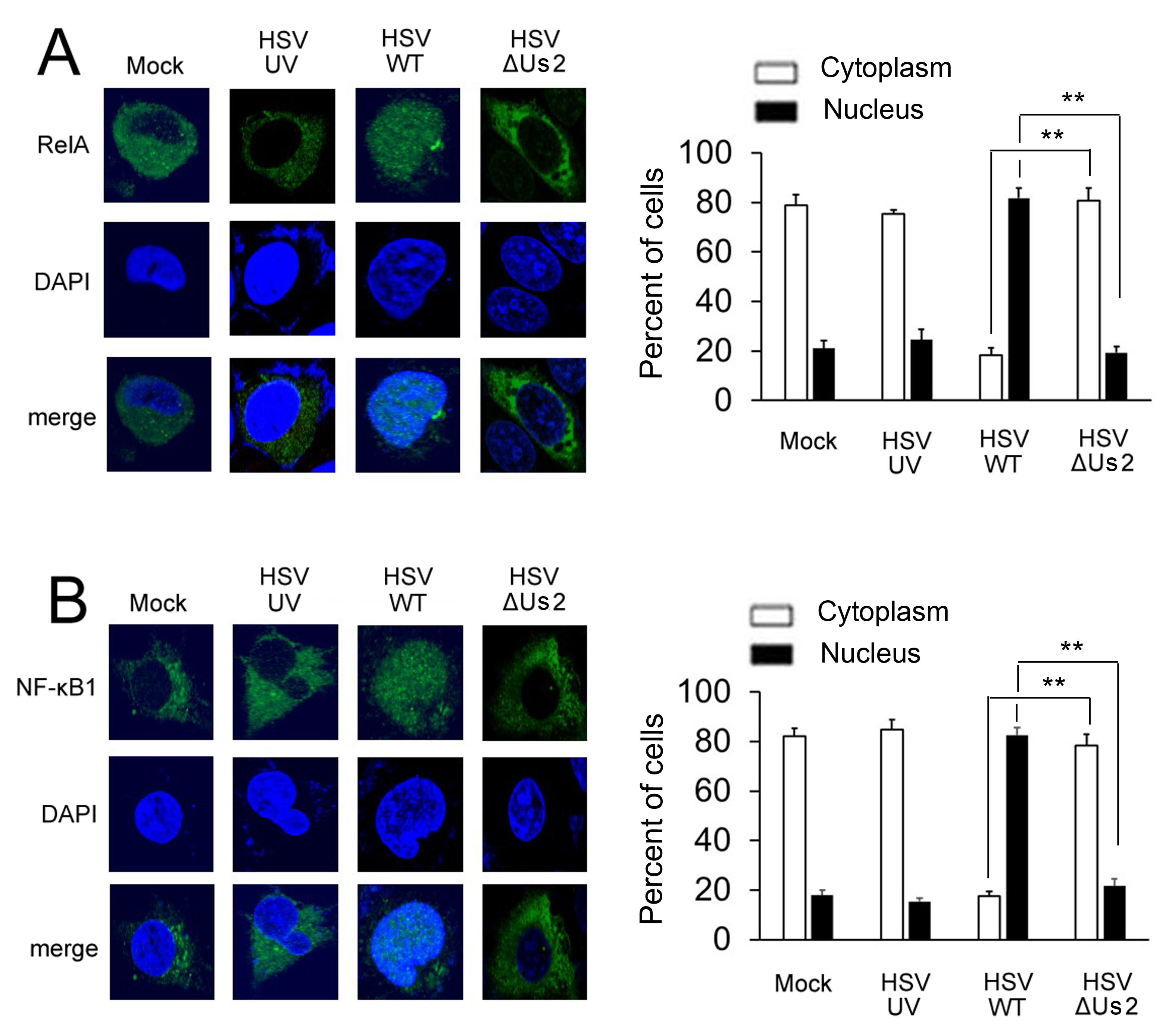


**Supplemental Figure 6**


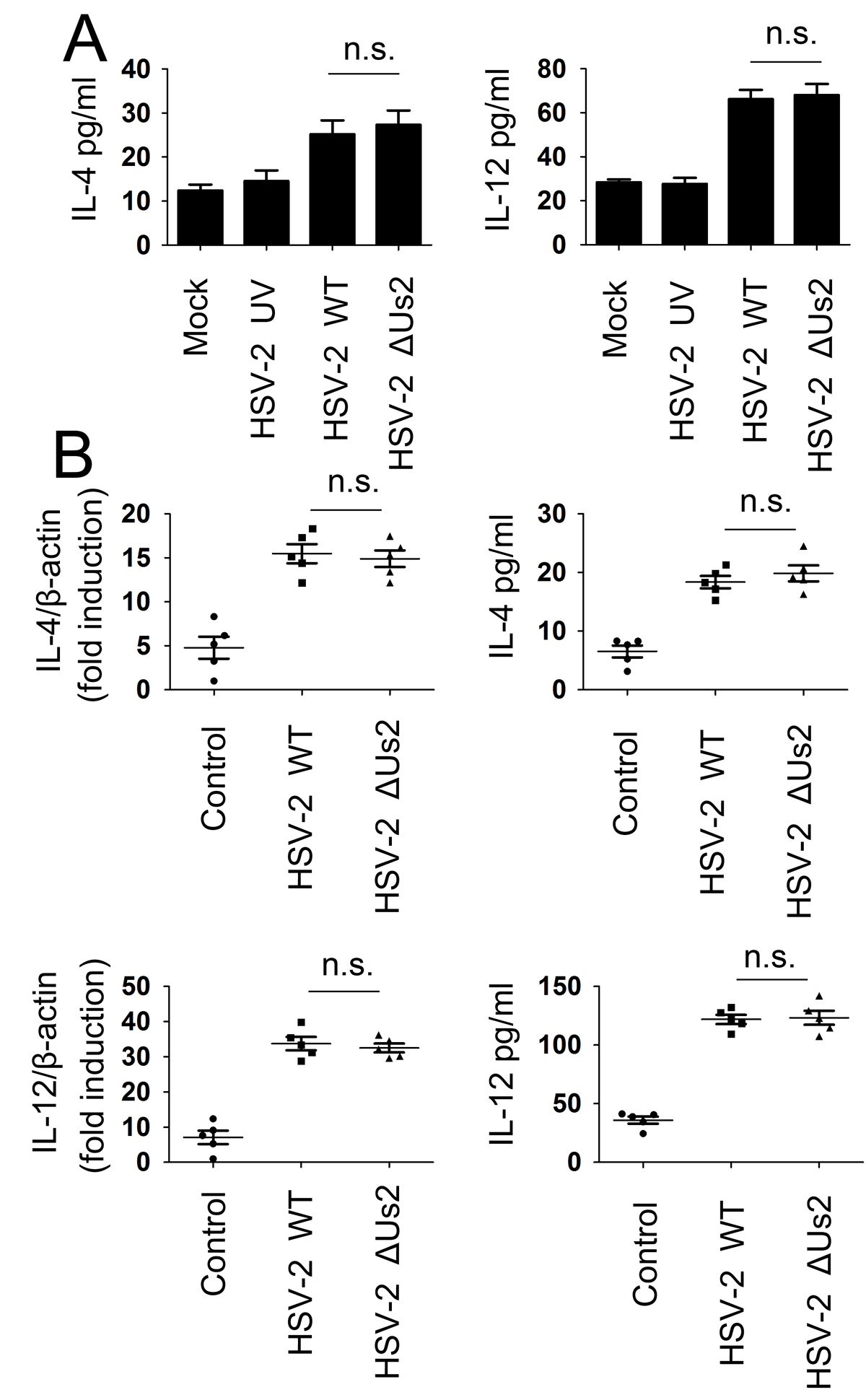


**Supplemental Figure 7**

**
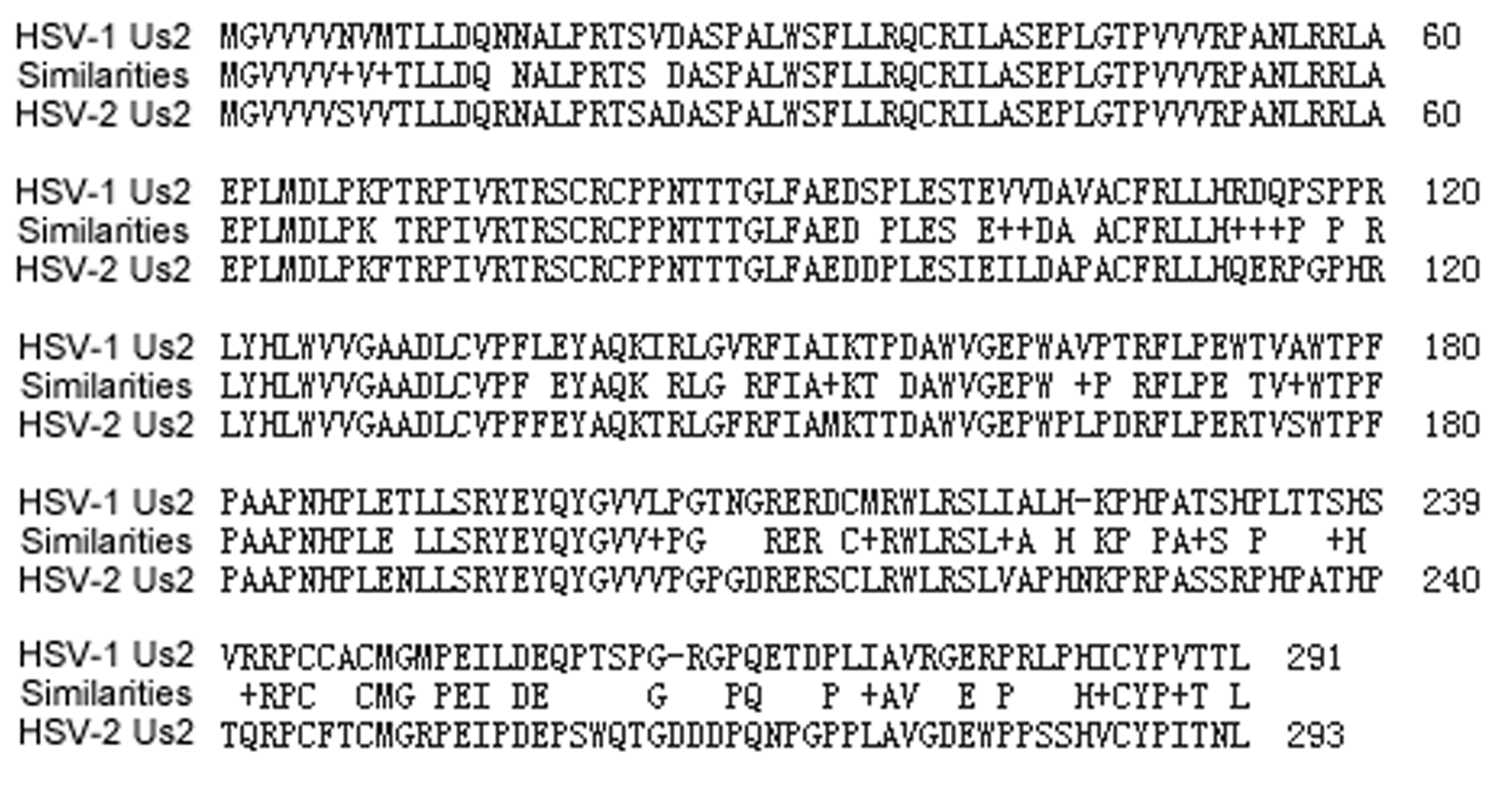
**

**Supplemental Table 1**: Primers Used in Real-time PCR.

| **Gene name** | **5’ primer** | **3’ primer** |
| --- | --- | --- |
| **β-actin (mouse)** | 5’-GCTGTCCCTGTATGCCTCTGGTC-3’ | 5’-CTTTGATGTCACGCACGATTTCC-3’ |
| **TNF-α (mouse)** | 5’-CGGAGTCCGGGCAGGTCTACTTT-3’ | 5’-GTCCAGGTCACTGTCCCAGCATC-3’ |
| **IL-6 (mouse)** | 5’-AGCCCACCAAGAACGATAGTCAA-3’ | 5’-CTCATTTCCACGATTTCCCAGAG-3’ |
| **IL-8 (mouse)** | 5’-GGCTTTGCGTTGATTCTGGGAACT-3’ | 5’-AGCGGTGTCCTGATTATCGTCCT-3’ |
| **CCL2 (mouse)** | 5’-CACTCACCTGCTGCTACTCATTC-3’ | 5’-ATTTACGGGTCAACTTCACATTC-3’ |
| **IFN-α (mouse)** | 5’-AGGAGCGTCTGGAAATACCTGTG-3’ | 5’-TTCGTTGGAATAGTTGCCCGAGT-3’ |
| **β-actin human)** | 5’-CCCCTCCATCGTCCACCGCAAAT CGG-3’ | 5’-GATGCTCGCTCCAACCGACTGCT -3’ |
| **TAK1 (human)** | 5’-CACAGACCAGCAGACAGGACAGA-3’ | 5’-TAAACGCTCCACAGAGGCAGAAT-3’ |

**Supplementary Table 2:** The target sequence of siRNAs

| siRNAs | Target sequence (5’ to 3’) |
| --- | --- |
| shRNA-TAK1 #1 | 5’-CATTTGATACCTTAGCTAAAGCACT-3’ |
| shRNA-TAK1 #2 | 5’-GCATTTGATACCTTAGCTAAAGCAC-3’ |
| shRNA-TAK1 #3 | 5’-ATTTGATACCTTAGCTAAAGCACTT-3’ |
| shRNA-control | 5’-GTTCTCCGAACGTGTCACGT-3’ |
